# EXAMINING THE ASSOCIATION OF HISTORICAL REDLINING WITH NURSING HOME QUALITY

**DOI:** 10.1093/geroni/igad104.3319

**Published:** 2023-12-21

**Authors:** Narcissa Plummer, Aneeka Ratnayake, Chelsea Wong, Brianne Olivieri-Mui

**Affiliations:** Northeastern University, Boston, Massachusetts, United States; Northeastern University, Boston, Massachusetts, United States; HebrewSenior Life, Boston, Massachusetts, United States; Northeastern University, Boston, Massachusetts, United States

## Abstract

**Aim:**

Redlining systemically relegated people of color to lower-resourced areas which are known to have had long-term effects on access to healthcare; however, it is not currently known if access to nursing home (NH) quality was impacted.

**Methods:**

In this cross-sectional descriptive study, we created a Geographical Information Systems map of the distribution of Massachusetts NH in the year 2021, layered over a 1934 redlining map with neighborhood categories: Best, Still Desirable, Declining, and Hazardous. Our dataset included redlining categories, 2021 NH characteristics (e.g., bed size, 5-star quality rating), and change in median household income from 1930 – 2021 (proxy for resources, in 2021 dollars). Descriptive statistics estimated the association of each variable with NHs’ 5-star quality rating.

**Results:**

The majority of NHs were in historically Declining (n=45,71%) or Hazardous (n=10,16%) neighborhoods. While 3% (n=2) were in Best neighborhoods. Both NHs in Best neighborhoods were 5-star, while 1 NH was 5-star in Hazardous neighborhoods. There were 63 NH included with a mean change in median household income of $47,684.06 (SD $37,844.01). The greatest increases in mean income were observed in hazardous neighborhoods containing 1-4-star NHs. The highest increase in mean income was $140,000 (n=1), occurring in a neighborhood with a 4-star NH.

**Conclusion:**

Despite the growth of resources in hazardous neighborhoods, most NHs in these areas were less than 4-stars. Increasing resources may not benefit NH quality in historically redlined/hazardous areas. Future research should examine this phenomenon at a national level.

